# Experimental Study on the Effects of Carbonated Steel Slag Fine Aggregate on the Expansion Rate, Mechanical Properties and Carbonation Depth of Mortar

**DOI:** 10.3390/ma17133279

**Published:** 2024-07-03

**Authors:** Pengfei Gao, Jian Wang, Jianjun Cui, Yongyu Yuan, Yuanyuan Song

**Affiliations:** 1 Inspection and Certification Co., Ltd. MCC, Beijing 100088, China; 2 School of Civil Engineering, Sun Yat-sen University, Guangzhou 510275, China; 3 Taishan Nuclear Power Joint Venture Co., Ltd., Taishan 529200, China; 4 School of Civil Engineering, Beijing Jiaotong University, Beijing 100044, China

**Keywords:** mortar, carbonated steel slag fine aggregate, expansion performance, mechanical property, carbonation performance

## Abstract

Steel slag is the main by-product of the steel industry and can be used to produce steel slag fine aggregate (SSFA). SSFA can be used as a fine aggregate in mortar or concrete. However, SSFA contains f-CaO, which is the main reason for the expansion damage of mortar and concrete. In this study, the carbonation treatment of SSFA was adopted to reduce the f-CaO content; the influence of the carbonation time on the content of f-CaO in the SSFA was studied; and the effects of the carbonated SSFA replacement ratio on the expansion rate, mechanical properties and carbonation depth of mortar were investigated through tests. The results showed that as the carbonation time increased, the content of f-CaO in the SSFA gradually decreased. Compared to the mortar specimens with carbonated SSFA, the specimens with uncarbonated SSFA showed faster and more severe damage and a higher expansion rate. When the replacement ratio of carbonated SSFA was less than 45%, the carbonated SSFA had an inhibitory effect on the expansion development of the specimens. The compressive strengths of the specimens with a carbonated SSFA replacement ratio of 60% and 45% were 1.29% and 6.81% higher than those of the specimens with an uncarbonated SSFA replacement ratio of 60% and 45%, respectively. Carbonation treatment could improve the replacement ratio of SSFA while ensuring the compressive strength of specimens. Compared with mortar specimens with uncarbonated SSFA, the anti-carbonation performance of mortar specimens with carbonated SSFA was reduced.

## 1. Introduction

Steel slag is an inevitable by-product in the steel production process, accounting for about 25% of the total steel production [1], and it is mainly divided into converter slag, open hearth furnace slag and electric arc furnace slag. In China, only about 30% of the steel slag is effectively utilized, lagging behind other developed countries [2,3], and the cumulative storage capacity of steel slag was 1.468 billion tons by 2020 [4]. Most steel slag is treated as industrial waste, and it not only occupies valuable land resources but also leaches out heavy metal ions (such as Zn, Pb, Cr, Ni, etc.), causing serious pollution to the surrounding environment [5,6,7]. Therefore, improving the utilization rate of steel slag is crucial.

To date, some scholars have conducted extensive research on the comprehensive utilization of steel slag [8,9,10,11,12]. The most common method is to use steel slag as a building material, such as directly as a roadbed cushion filler, finely ground as a cementitious material or crushed and screened as an aggregate [13]. Compared with traditional aggregates, the utilization of steel slag as an aggregate is advantageous [14,15]. Faraone et al. [16] found that, with a sufficient water–cement ratio and cement–slag ratio and an appropriate particle size of steel slag, the mortar could exhibit good compressive strength when cured for 28 days. Pellegrino et al. [17] used a large amount of oxidizing electric arc furnace (EAF) slag instead of natural sand and natural gravel to pour concrete, and they discovered that using EAF slag as a coarse aggregate helped to improve the compressive strength, tensile strength and elastic modulus, but it might have a negative impact on the compressive strength when used as a fine aggregate. Devi et al. [18] discussed the optimal dosage of steel slag as coarse and fine aggregates and showed that the mechanical properties of concrete with the addition of steel slag under the optimal dosage were better than those of ordinary concrete. However, the free calcium oxide (f-CaO) and free magnesium oxide (f-MgO) contained in steel slag undergo slow hydration to form calcium hydroxide (Ca(OH)_2_) and magnesium hydroxide (Mg(OH)_2_), with their volumes increasing by 1.98 times and 2.48 times, respectively, which may lead to problems related to poor volume stability, such as the cracking of concrete [19,20]. Therefore, caution should be applied when using steel slag as an aggregate [21,22]. Meanwhile, some studies have shown that the hydration expansion of f-MgO within a certain threshold can compensate for the shrinkage of steel slag products, which is beneficial to their durability [23,24]. Therefore, it is generally believed that f-CaO is the main reason for the poor stability of steel slag [25], and the question of how to eliminate or weaken the expansion effect caused by f-CaO is also the key point of research on the stability of steel slag.

Meanwhile, the use of steel slag to capture CO_2_ and generate stable carbonates has become a research focus in recent years [26,27,28]. Wang et al. [29] summarized the basic principles and common methods of steel slag carbonation and analyzed the effects of the temperature, liquid–solid ratio, carbonation time, CO_2_ concentration and pressure and other factors on the carbonation effect of steel slag. Liu et al. [30] and Chen et al. [31] carbonized steel slag used as a supplementary cementitious material through direct carbonation and indirect carbonation, respectively, and both found that carbonation optimized the pore structure of the cement matrix and thus increased the compressive strengths of the mortars. Pang et al. [32] compared the basic characteristics of natural fine aggregate, steel slag aggregate and carbonated steel slag aggregate and found that the compressive strength of concrete with carbonated steel slag fine aggregate could be effectively improved. However, Yu et al. [33] showed different results, setting up four groups of mortar specimens with carbonated steel slag replacement ratios of 0%, 15%, 30% and 45% and finding that the compressive strengths of mortar specimens with carbonated steel slag at 28 days were always smaller than those of mortar specimens without steel slag aggregate. Thus, research on carbonated steel slag mainly focuses on its use as a cementitious material, and the conclusions about the mechanical properties of mortar specimens with carbonated steel slag fine aggregate are not unified. Moreover, due to the significant differences in the physical properties and chemical compositions of different types of steel slag [34,35], there is great uncertainty regarding the expansion and long-term performance of mortar specimens with carbonated steel slag fine aggregate.

To fill this research gap, the influence of carbonated steel slag fine aggregate (SSFA) on the properties of mortar was investigated in this study. Firstly, the ethylene glycol–TG method was used to measure the content of f-CaO in SSFA, and the effect of the carbonation time on the content of f-CaO was studied. Then, the effects of different carbonated SSFA replacement ratios on the expansion performance, mechanical properties and carbonation performance of the mortar were compared and discussed.

## 2. Experimental Program

### 2.1. Raw Materials

The cement used was ordinary Portland cement with a strength grade of 42.5 R, and the chemical composition is shown in Table 1. The fine aggregates included natural sand and steel slag, among which the natural sand was well-graded natural river sand. Steel slag was taken from a steel plant and crushed by a jaw crusher. The SSFA with a particle size of 0.15~5.0 mm was obtained by a ball crusher. The mixing water was tap water.

### 2.2. Carbonation of SSFA

**Figure 1:**
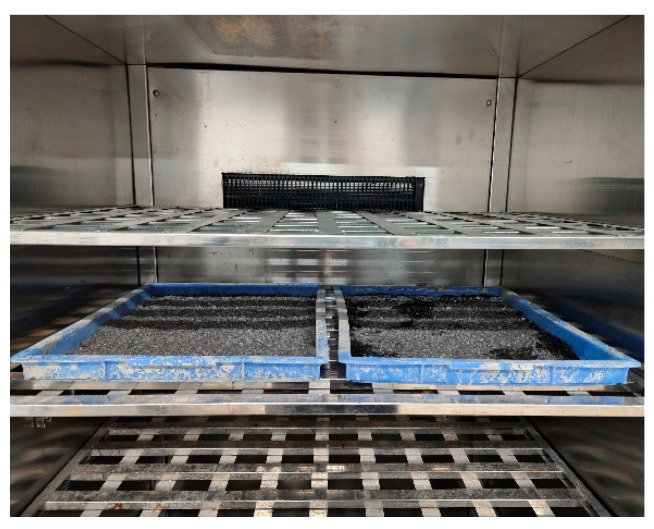
Carbonated SSFA.

The prepared SSFA was laid flat on a plastic tray and placed into a concrete carbonation chamber for rapid carbonation, as shown in Figure 1. CO_2_ gas with purity of 99% was injected into the carbonation chamber. According to the Chinese Standard GB/T 50082-2009 [36], the conditions inside the carbonation chamber included a temperature of 20 °C, relative humidity of 70% and a CO_2_ concentration of 20%. To ensure complete carbonation, the SSFA was taken out from the chamber after 7 days. The carbonation effect of the SSFA was quickly determined by the color change of a phenolphthalein solution, and the results indicated that the carbonation of SSFA was complete.

### 2.3. Mortar Specimens

Seven groups of mortar specimens were used for the expansion and compression tests, as displayed in Table 2. The water–cement ratio was 0.47, and the mass ratio of cement to sand was 1:2.25. Additionally, Table 3 shows the grading of the experimental sand, and the corresponding SSFA was used to replace the natural river sand according to the chosen replacement ratios. To improve the reliability of the experimental data, there were three mortar bars used in the expansion test and three cubes used in the compression test for each group, with a mortar bar size of 25 × 25 × 280 mm and a cube size of 40 × 40 × 40 mm. The finished mortar bars were placed into a standard curing room (90% relative humidity and 20 ± 2 °C temperature) with molds and demolded after being cured for 24 h, while the cubes were cured for 28 days after being demolded.

Seven groups of specimens were also used for the carbonation test, as listed in Table 4. The chosen water–cement ratio was 0.50, and the mass ratio of cement to sand was 1:3. The fineness modulus of the natural sand was 3.06. Table 5 shows the continuous grading of the SSFA. There were three cubes for each group, with a size of 100 × 100 × 100 mm. After being poured and shaped, all specimens were demolded and placed into a standard curing room with 90% relative humidity and a temperature of 20 ± 2 °C for 28 days.

Among all specimens tabulated in Table 2 and Table 4, EM represents the groups used for the study of the expansion performance and mechanical properties of the specimens, while CR refers to those groups used to study the carbonation performance of the specimens. The middle number denotes the replacement ratio of SSFA in this group of specimens. The letters C and UC indicate whether carbonated SSFA or uncarbonated SSFA was used in this group of specimens, respectively.

### 2.4. Determination of f-CaO Content in SSFA

To investigate the effect of the carbonation time on the content of f-CaO in SSFA, the content of f-CaO was measured at different carbonation times according to Chinese Standard YB/T 4328-2012 [37]. Approximately 10 g of SSFA was sealed and stored every 2 h until the content of f-CaO was measured to be below 1.00%.

The content of f-CaO in SSFA was determined by the ethylene glycol–TG method. First of all, an ethylene glycol calcium solution was obtained by reacting an ethylene glycol solution with f-CaO, and then an EDTA-2Na solution was used to titrate the ethylene glycol calcium solution, so that the total free calcium content (*c*_1_) in the SSFA could be obtained through Equation (1). Subsequently, based on the thermal decomposition characteristics of Ca(OH)_2_ under the condition of a high temperature, the content of Ca(OH)_2_ (*c*_2_) in the SSFA was measured using a thermogravimetric analyzer. Finally, the difference between *c*_1_ and *c*_2_ indicated the content of f-CaO.

(1) $$c_{1}=\frac{T_{\mathrm{CaO}}\times V}{m\times 1000}\times 100\%$$

where *T*_CaO_ is the mass fraction of total free calcium (%), calculated from *T*_CaO_ = *c*(EDTA)·56.08; *c*(EDTA) is the concentration of the EDTA standard titration solution (mol/L); *V* is the volume of the EDTA standard titration solution (mL); and *m* is the mass of the steel slag sample (g).

### 2.5. Expansion Test of Mortar Specimens

According to the Chinese standard JGJ 52-2006 [38], the volume stability of the mortar specimens was studied through the alkali activity test (rapid method) using crushed stones or pebbles. After demolding, the specimens were immersed in a curing tube and cured in a water bath at 80 °C for 24 h. The initial lengths (*l*_0_) of the specimens were measured, and then the specimens continued to be immersed in the curing tube, filled with 1 mol/L NaOH solution with a temperature of 80 °C. Starting from the day on which the initial lengths were measured, the changes in the specimens were observed at 3, 7, 14, 21, and 28 days, and the corresponding lengths (*l_i_*) were measured. The formula for the calculation of the expansion rates of the specimens is given in Equation (2), and the average value of the expansion rates of the three specimens was regarded as the result for each group of specimens.

(2) $$\varepsilon_{i}=\frac{l_{i}-l_{0}}{l_{0}-\left(\Delta_{1}+\Delta_{2}\right)}\times 100\%$$

where *ε_i_* is the expansion rate of a specimen at the *i*-th day (%); *l_i_* is the length of a specimen at the *i*-th day (mm); *l*_0_ is the initial length of a specimen (mm); and Δ_1_ and Δ_2_ are the lengths of the measuring heads at the left and right ends of the specimen (mm).

### 2.6. Compression Test of Mortar Specimens

According to the Chinese standard GB/T 17671-2021 [39], the compressive strengths of the mortar specimens were determined. The specimen was placed in the pressure testing machine and uniformly loaded at a rate of 2.4 kN/s until failure. The average value of the compressive strength of the three specimens was regarded as the result for each group of specimens.

### 2.7. Carbonation Test of Mortar Specimens

**Figure 2:**
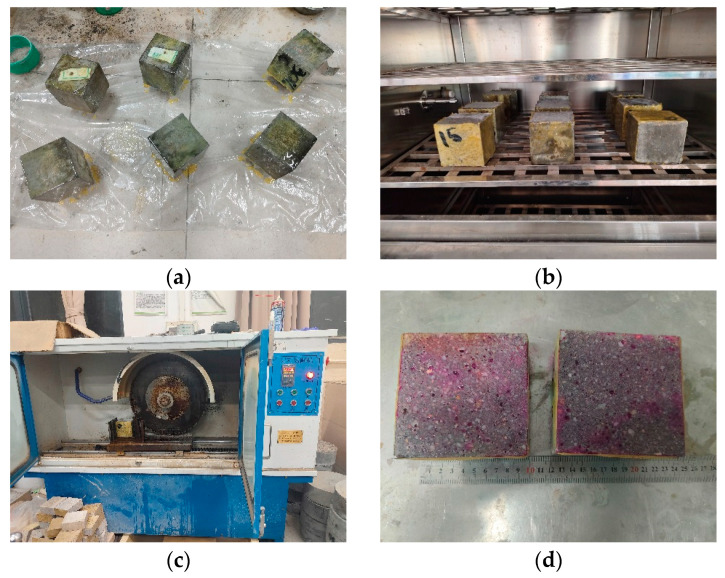
Carbonation test. (**a**) Coating cubes with epoxy resin; (**b**) carbonation; (**c**) cutting along the midline; (**d**) spraying phenolphthalein solution.

Figure 2 shows the process of the carbonation test. To ensure the one-dimensional carbonation of the specimens, only one side of the specimens was retained as the CO_2_ erosion surface, while the other five surfaces were coated with epoxy resin. According to the Chinese standard GB/T 50082-2009 [36], the specimens were placed in batches in the carbonation chamber for the carbonation test, and the conditions in the chamber were consistent with the carbonation environment described in Section 2.2.

After 7 days of carbonation, the specimens were taken out and cut along the midline using a rock cutting machine. A phenolphthalein solution with a concentration of 1% was sprayed onto the cutting surface, and, after about 30 s, the carbonation depth along the length of the cutting surface was measured every 10 mm. There was a total of 10 measurement points on the cutting surface of each specimen, and the average value of the carbonation depths of the three specimens was calculated as the carbonation depth for each group of specimens.

## 3. Results and Discussion

### 3.1. Effect of Carbonation Time on Content of f-CaO in SSFA

**Figure 3:**
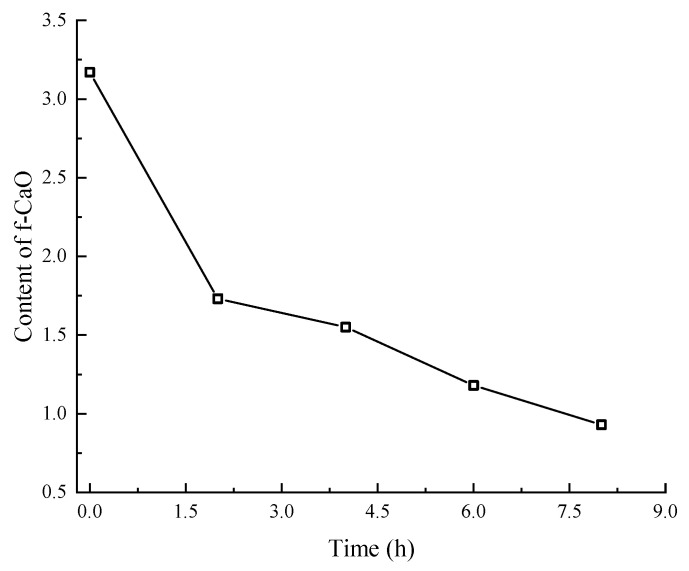
The f-CaO content in SSFA with the carbonation time.

The content of f-CaO in SSFA at different carbonation times is shown in Figure 3. It is obvious that as the carbonation time increases, the content of f-CaO gradually decreases. After 8 h of carbonation, the content of f-CaO drops to 0.93%, only 29.34% of the initial content, which indicates that the carbonation of steel slag can effectively reduce the f-CaO content. The final products of carbonation are mainly CaCO_3_ crystals with stable chemical properties, which can fill the gaps on the surface of the SSFA and consolidate the original skeleton of the SSFA, thereby helping to improve the strength and volume stability of the SSFA.

From Figure 3, it can also be observed that the decrease rate of the f-CaO content with the carbonation time slows down. In the first two hours, the content of f-CaO reduces from 3.17% to 1.73%, with a decrease of 45.43%, and then the decrease in the content of f-CaO between adjacent carbonation times does not exceed 25%. This may be because the products of early carbonation are deposited on the surface of the steel slag, which, to some extent, hinders the diffusion of CO_2_ gas into the interior of the steel slag, causing a slowdown in the carbonation reaction [40].

### 3.2. Expansion Rate of Mortar Specimens

**Figure 4:**
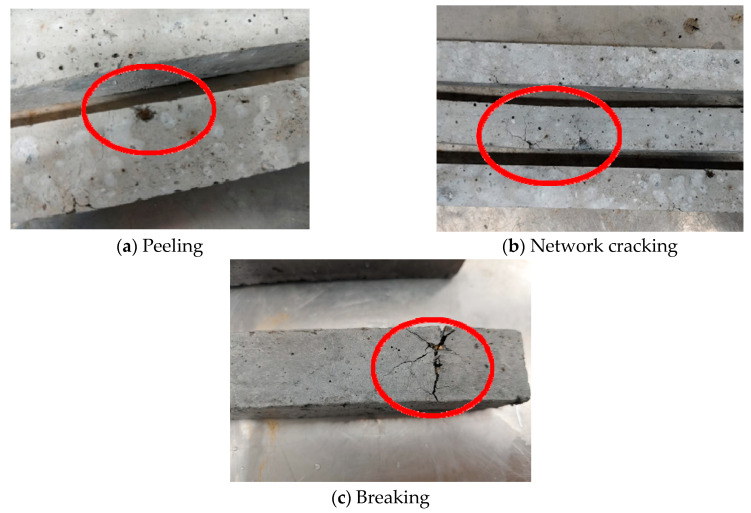
Expansion damage phenomena of specimens.

While recording the changes in the expansion rate of the specimens at different ages, the expansion phenomena can also be observed. As shown in Figure 4, there is certain regularity in the surface damage of each group of specimens. The surfaces of the specimens first show a peeling phenomenon, the surface concrete falls off in powder form, brown explosion points appear, and the distributions of the peeling positions and the explosion points are scattered and have no obvious law. Subsequently, several tiny cracks appear around the explosion points, and, as the experimental time increases, the cracks develop from the points to the surrounding areas, forming a network distribution. Finally, the width of the cracks gradually increases, and the specimens break along the cracks.

**Figure 5:**
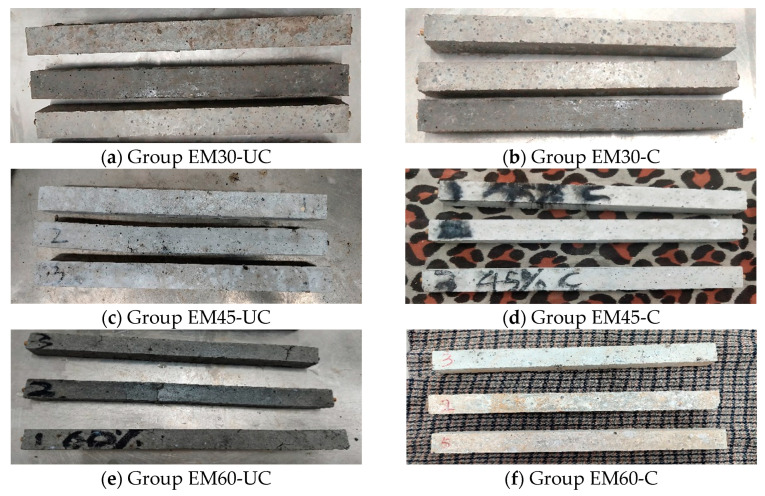
Expansion test at 14 days.

However, the rate of surface damage development varies among different groups of specimens. At the beginning of the expansion test, there is no clear damage to any group of specimens. When the experiment lasts for 14 days, the specimens in both Groups EM60-C and EM60-UC (Figure 5e,f) show several brown explosion points and obvious network cracks on the surface, while the specimens in Group EM60-UC have more severe damage, with longer and wider cracks, and some cracks reach a width of 0.2 mm. Although the specimens in Group EM45-UC (Figure 5c) have one or two brown explosion points on the surface, the overall damage is not significant, while other groups of specimens (Figure 5a,b,d) have no damage. When the experiment lasts for 21 days, multiple breaks occur on the surfaces of the specimens in Group EM60-UC, indicating the end of the expansion test. One specimen in Group EM60-C also breaks into several parts, while the network cracks on the surface of the remaining specimens continue to develop. The specimens in Groups EM45-C and EM45-UC show obvious peeling and cracking phenomena, while the other three groups of specimens still show little damage. When the experiment reaches 28 days, there is still no obvious damage on the surfaces of the specimens in Groups EM0 and EM30-C, while a small number of cracks appear on the surfaces of the specimens in both Groups EM30-UC and EM45-C. The network cracking of the specimens in Group EM45-UC and the remaining specimens in Group EM60-C becomes increasingly obvious and the cracks widen, but no break occurs.

It is not difficult to find that, compared to the carbonated SSFA specimens, the uncarbonated SSFA specimens show faster and more severe damage, demonstrating that the carbonation of SSFA can significantly improve the volume stability of the specimens. Moreover, the expansion damage of the specimens becomes increasingly severe with the increase in the SSFA replacement ratio, which means that the SSFA replacement ratio does indeed have a significant impact on the volume stability of the specimens.

**Figure 6:**
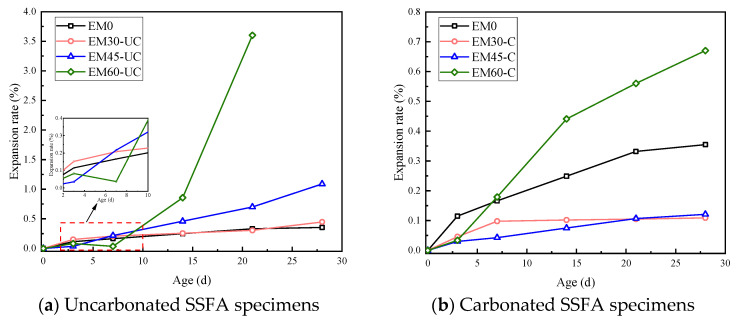
Change in expansion rate with age of specimens.

The expansion rate varies with the age of the specimens, as shown in Figure 6. Comparing Figure 6a with Figure 6b, it can be seen that, under the same conditions, the expansion rate of the uncarbonated SSFA specimens is much higher than that of the carbonated SSFA specimens, illustrating that the carbonation treatment of SSFA is beneficial in improving the volume stability of the specimens. According to Section 3.1, the content of f-CaO in SSFA significantly decreases due to carbonation, and its hydration expansion effect is weakened. Moreover, the CaCO_3_ crystals generated by carbonation will fill the pores on the surface or wrap the SSFA, which has a certain hindering effect on the subsequent hydration process. Therefore, the expansion rate of the carbonated SSFA specimens is generally small.

Figure 6a shows that the early expansion rate of the uncarbonated SSFA specimens fluctuates around 0.10%, and the difference is not obvious. As the age increases, the expansion rates of the specimens in Groups EM30-UC and EM45-UC change relatively smoothly. The expansion rate of the specimens in Group EM30-UC is nearly identical to that of the specimens in Group EM0, and the difference in the expansion rates between the two groups at the same age is within 0.100%. Moreover, the expansion rate of the specimens in Group EM60-UC is much higher than that of the others, with an expansion rate of 0.857% at the age of 14 days, which is 234.8% and 86.7% higher than that of the specimens in Groups EM30-UC and EM45-UC, respectively, and the expansion development is also faster, with breaks appearing first. Therefore, the expansion rate of the uncarbonated SSFA specimens increases with the replacement ratio. When the replacement ratio of SSFA is high, the development of the expansion rate of the SSFA specimens can be divided into several stages. In the early stage, the hydration reaction of the specimens is mainly that of a cement-based material, and the expansion development is slow, with little difference in the change in the expansion rate. After the basic hydration of the cementitious materials is completed, the SSFA in the specimens begins to slowly hydrate, and the expansion effect begins to appear. As time passes, the expansion effect generated by the hydration of SSFA gradually becomes apparent, and the expansion rate of the specimens continues to increase. When the accumulated expansion stress inside the specimens exceeds the tensile strength of the matrix, micro-cracks will appear around the steel slag particles. If the steel slag is located near the surfaces of the specimens, it is easy to see the peeling phenomenon and explosion point damage on the surfaces of the specimens.

Figure 6b shows that, for the carbonated SSFA specimens, the expansion rates on the third day are slower than those of the specimens without SSFA. Furthermore, during the experiment, the expansion rates of the specimens in Groups EM45-C and EM30-C are always slower than those of the specimens in Group EM0, illustrating that when the replacement ratio of carbonated SSFA is less than 45%, the carbonated SSFA has an inhibitory effect on the expansion development of the specimens. This is probably because carbonation treatment mainly involves a reaction with the f-CaO in the steel slag to generate CaCO_3_ crystals, and when carbonated steel slag is used as a fine aggregate to replace some natural sand, not only does it weaken the expansion effect of the steel slag itself, but it also improves the strength of the mortar, making the specimens less prone to expansion damage. In addition, the expansion rates of the specimens in Group EM60-C are nearly always larger than those of the specimens in Group EM0, with the expansion rate at the 28th day being 88.79% higher than that of EM0, demonstrating that the replacement ratio of carbonated SSFA has an important influence on the expansion rate of the specimens. Although carbonation accelerates the hydration of the f-CaO in the SSFA and helps to mitigate the problem of poor volume stability, the total amount of SSFA used increases, and the superimposed expansion effect is also significant, resulting in a much higher expansion rate.

### 3.3. Compressive Strength of Mortar Specimens

**Figure 7:**
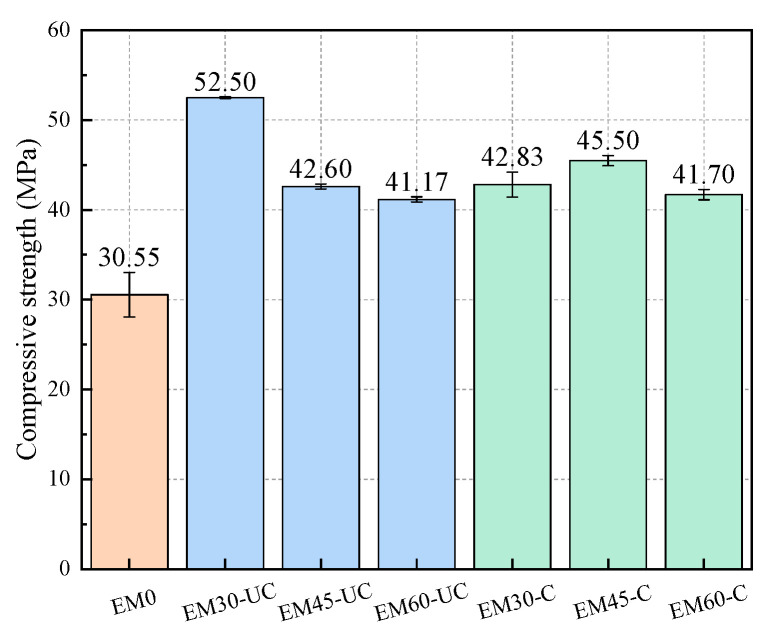
Compressive strength of specimens.

Figure 7 shows the compressive strength of the specimens. It is obvious that the compressive strength of the specimens with SSFA is higher than that of the ordinary specimens (30.55 MPa), i.e., the addition of SSFA is beneficial for the compressive strength of the specimens. This may be because, compared to natural sand with a smooth surface, steel slag, whose surface is quite rough, can better blend with cement slurry. In addition, the hydration product of f-CaO in SSFA could fill the pores inside the mortar, improving the compactness of the mortar, which is manifested as an improvement in the compressive strength of the specimens.

When the replacement ratio of SSFA is the same, the compressive strengths of the specimens in Groups EM60-C and EM45-C are 1.29% and 6.81% higher than those of EM60-UC and EM45-UC, respectively, which indicates that the carbonation of SSFA can have a positive effect in terms of the improvement of the compressive strength of the specimens. This may be related to the pre-filling of the pores with CaCO_3_ crystals, as described in Section 3.1, making the structure of the steel slag particles more compact.

For the specimens with uncarbonated SSFA, the compressive strength of the specimens in Group EM60-UC is the lowest; specifically, it is 21.58% and 3.36% lower than that of the specimens in Groups EM30-UC and EM45-UC, respectively. In other words, the compressive strength of the specimens decreases with the increase in the replacement ratio of SSFA. This is because, although uncarbonated SSFA can have a positive effect on the compressive strength of the specimens, the expansion effect generated by the hydration of SSFA gradually becomes apparent with the increase in the replacement ratio of SSFA, manifesting as a decrease in the compressive strength of the specimens. For the specimens with carbonated SSFA, the specimens in Group EM45-C have the highest compressive strength, which is 6.23% and 9.11% higher than that of the specimens in Groups EM30-C and EM60-C, respectively. It is evident that carbonation treatment can improve the replacement ratio of SSFA while ensuring the compressive strength of the specimens.

### 3.4. Carbonation Depth of Mortar Specimens

**Figure 8:**
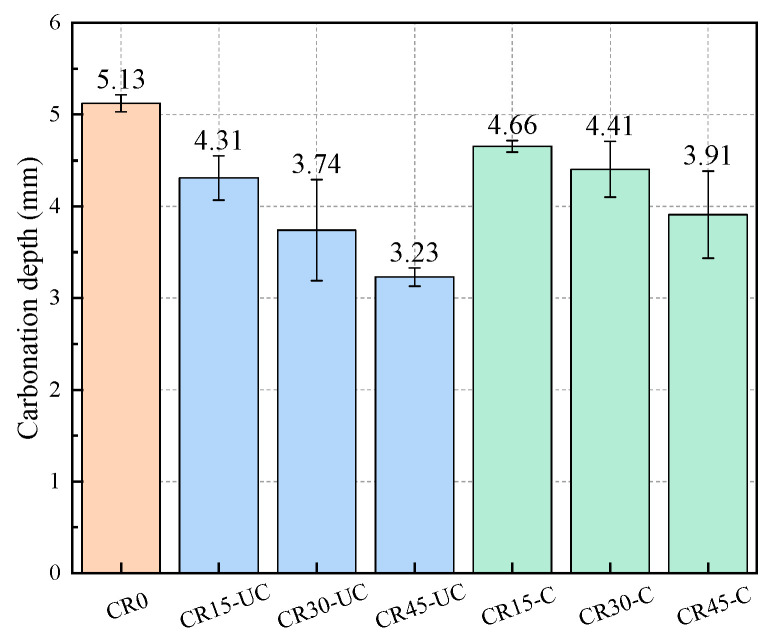
Carbonation depth of specimens.

From Figure 8, it can be seen that, compared to the specimens in Group CR0, the carbonation depths of the specimens in Groups CR15-UC, CR30-UC and CR45-UC decrease by 15.98%, 27.10% and 37.04%, respectively, while the carbonation depths of the specimens in Groups CR15-C, CR30-C and CR45-C are reduced by 9.36%, 14.23% and 23.78%, respectively, indicating that the carbonation resistance of the specimens with SSFA is better than that of the ordinary specimens. In addition, as the replacement ratio of SSFA increases, the carbonation depth of the specimens with uncarbonated SSFA significantly decreases, showing that the carbonation resistance of the specimens improves with the increase in the replacement ratio, when the replacement ratio of SSFA is less than 45%. The reasons for this phenomenon are that the f-CaO contained in SSFA slowly hydrates during the carbonation test, requiring the absorption of CO_2_ gas, and, at the same time, the Ca(OH)_2_ or CaCO_3_ produced by hydration can fill the surrounding pores, preventing CO_2_ gas from continuing to diffuse into the interiors of the specimens. Therefore, the carbonation resistance of specimens with SSFA is better, and the improvement in the carbonation resistance is more significant with the increase in the replacement ratio of SSFA.

Compared with the specimens with uncarbonated SSFA, the carbonation depth of the specimens with carbonated SSFA is slightly larger, but the difference in the carbonation depth between the two types of specimens with the same replacement ratio of SSFA does not exceed 18%. The reason for this small difference is that the content of f-CaO in the SSFA after carbonation visibly drops, and the absorption capacity of CO_2_ is weakened; hence, the anti-carbonation performance of the specimens with carbonated SSFA reduces slightly.

## 4. Conclusions

This paper first explored the influence of the carbonation time on the content of f-CaO in SSFA and then investigated the influence of carbonated SSFA on the expansion performance, mechanical properties and carbonation performance of mortar specimens, and the following conclusions can be drawn.

(1) The carbonation treatment of steel slag can effectively reduce the f-CaO content. After 8 h of carbonation, the content of f-CaO in SSFA drops from 3.17% to 0.93%, only 29.34% of the initial content.
(2) Compared to the mortar specimens with carbonated SSFA, the specimens with uncarbonated SSFA shows faster and more severe damage and a higher expansion rate. When the replacement ratio of carbonated SSFA is less than 45%, the carbonated SSFA has an inhibitory effect on the expansion development of the specimens. The carbonation treatment of SSFA can improve the replacement ratio of SSFA while ensuring the same volume stability of the mortar specimens.
(3) The compressive strength of mortar specimens with uncarbonated SSFA reduces with the increase in the replacement ratio of SSFA. The compressive strengths of the specimens with carbonated SSFA replacement ratios of 60% and 45% are 1.29% and 6.81% higher than those of the specimens with uncarbonated SSFA replacement ratios of 60% and 45%, which indicates that the carbonation of SSFA can have a positive effect in terms of the improvement in the compressive strength of the specimens. Carbonation treatment can improve the replacement ratio of SSFA while ensuring the compressive strength of specimens.
(4) Mortar specimens with SSFA have better carbonation resistance. When the replacement ratio of SSFA is less than 45%, the carbonation depth of the specimens significantly decreases with the increase in the replacement ratio. Compared with mortar specimens with uncarbonated SSFA, the carbonation depth of mortar specimens with carbonated SSFA is slightly larger, and their anti-carbonation performance is reduced.
(5) Carbonation treatment is a beneficial method to improve the stability of SSFA and provide guidance for the future application of mortar. Further study regarding the effect of carbonated steel slag coarse aggregate on the mechanical and expansion properties of concrete is recommended.

## Figures and Tables

**Table 1:** Chemical composition of cement (%).

SiO_2_	Al_2_O_3_	Fe_2_O_3_	CaO	MgO	SO_3_	Loss
22.25	4.98	3.47	64.84	1.51	1.83	1.12

**Table 2:** Quantities of mortar specimens in expansion and compression tests.

Group Number	Expansion Test	Compression Test	Replacement Ratio of SSFA (%)	SSFA
EM0	3	3	0	—
EM30-UC	3	3	30	Uncarbonated
EM30-C	3	3		Carbonated
EM45-UC	3	3	45	Uncarbonated
EM45-C	3	3		Carbonated
EM60-UC	3	3	60	Uncarbonated
EM60-C	3	3		Carbonated

**Table 3:** Grading of experimental sand.

Aperture Size of Test Sieves (mm)	4.75~2.36	2.36~1.18	1.18~0.60	0.60~0.30	0.30~0.15
Content (%)	10	25	25	25	15

**Table 4:** Groups of specimens in carbonation test.

Group Number	Replacement Ratio of SSFA (%)	SSFA
CR0	0	—
CR15-UC	15	Uncarbonated
CR15-C		Carbonated
CR30-UC	30	Uncarbonated
CR30-C		Carbonated
CR45-UC	45	Uncarbonated
CR45-C		Carbonated

**Table 5:** Grading of SSFA.

Aperture Size of Test Sieves (mm)	>4.75	4.75~2.36	2.36~1.18	1.18~0.60	0.60~0.30	0.30~0.15	<0.15
Content (%)	3.70	13.34	24.44	30.37	22.22	5.93	0

## Data Availability

The raw data supporting the conclusions of this article will be made available by the authors on request.
